# 35+ years of the additional singleton task: Design features and guidelines

**DOI:** 10.3758/s13414-026-03259-y

**Published:** 2026-04-28

**Authors:** Jan Theeuwes

**Affiliations:** 1 Cognitive Psychology, Vrije Universiteit Amsterdam, Amsterdam, Netherlands; 2 Institute Brain and Behavior Amsterdam (iBBA), Amsterdam, Netherlands; 3 Mind, Brain and Behaviour Research Centre (CIMCYC), Granada, Spain; 4 Zhejiang University, Hangzhou, Zhejiang China; 5 Department of Experimental and Applied Psychology, Vrije Universiteit Amsterdam, Van der Boechorststraat 1, 1081 BT Amsterdam, The Netherlands

**Keywords:** Attentional capture, Bottom-up attention, Top-down attention, Visual search

## Abstract

The additional singleton paradigm, introduced in the early 1990s, has become a cornerstone in attention research and the study of attentional capture. In this task, observers search for a unique target singleton while an irrelevant, but salient distractor singleton is also present. Decades of research have demonstrated that such distractors reliably slow responses, supporting a stimulus-driven account of attentional selection. This paper reviews the origins of the paradigm, key findings, and ongoing debates, with particular focus on design features that shape results. Critical factors include the consistency of target–distractor assignments, distractor prevalence, display size, target–distractor similarity, and the use of compound versus simple search tasks. Guidelines are presented to maximize the paradigm’s utility and to avoid misinterpretation of attentional capture effects. The review concludes that the additional singleton task continues to provide unique leverage in distinguishing stimulus-driven selection from top-down control.

While completing my PhD in the early 1990s, I introduced a paradigm that would later prove highly influential, ultimately contributing to the emergence of *attentional capture* as a distinct research field (Theeuwes, 1991, 1992, 1994b). Several years later, Simons (2000) coined the term *additional singleton paradigm* to describe the task, a name that has since become widely adopted (Liesefeld et al., 2024). Now more than 35 years old, this paradigm remains a cornerstone in attention research and continues to be widely used. The paper describing this paradigm has been cited over 2,250 times (Google Scholar), with its highest annual citation count occurring in 2024. In the current paper, I reflect on the origins of the paradigm, some of the controversies, and highlight key design considerations that researchers should keep in mind when employing the task. In this review, I will only focus on behavioral findings; other reviews also cover neuroimaging measures (e.g., Luck et al., 2021).

## The basic paradigm, Theeuwes (1991, 1992)

### The origin of the paradigm

When I developed the additional singleton paradigm, I was still working at the TNO Human Factors Research Institute, a research organization specializing in applied research for industry, private companies, and government agencies. At the time, I was part of the Traffic Behavior group, primarily conducting research on perception and attention in relation to traffic behavior. My main focus was on how road design influences driver behavior.

One day, during a discussion with engineers from the Department of Transportation, one of them posed a simple yet intriguing question: If two very salient traffic signs are placed along the road, which one will road users look at first? I found this to be a fascinating question, but I could not find a clear or direct answer in either the basic or applied research literature.

Although I did not yet hold a PhD, I recognized that this practical question could be reframed as a fundamental research problem—one that aligned with the theoretical framework of feature-based attention as described by Treisman (Treisman & Gelade, 1980). This line of thinking ultimately led to the development of the additional singleton paradigm.

### Basic task and findings

**Fig. 1:**
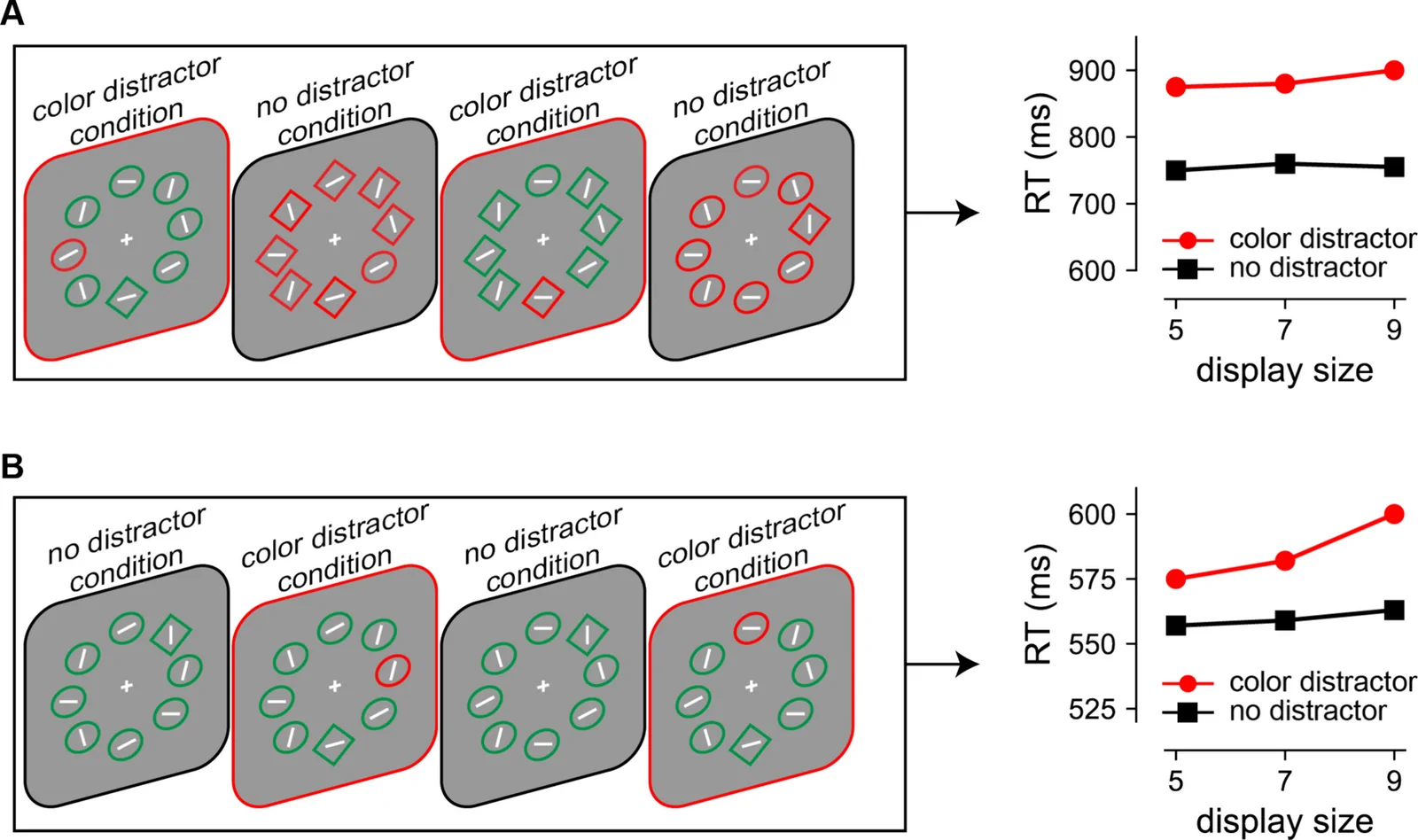
Stimuli and results from the additional singleton paradigm (Theeuwes, 1991, 1992). In version **A**, participants searched for a diamond among circles or a circle among diamonds, with the target shape changing randomly across trials. In version **B**, participants consistently searched for a green diamond among green circles. Observers responded by indicating the orientation (horizontal or vertical) of the line within the uniquely shaped target (e.g., a diamond among circles). The presence of a color distractor singleton (an item with a unique color) resulted in an increase in RT, serving as evidence of attentional capture. Despite the consistent search for a specific target (in **A**, a unique shape; in** B**, a green diamond), the attention of the observers was invariably drawn to the salient color distractor. (Figure adapted from Theeuwes, 2025) (Color figure online)

In the 1991 additional singleton task version of this task (Theeuwes, 1991) participants search for a unique shape singleton (a diamond between circles or a circle between diamonds varied within blocks) while a salient irrelevant color singleton (a single green item between red items or a red item between green items) is simultaneously present (see Fig. 1). In the 1992 version of this task (Theeuwes, 1992), observers always search consistently throughout the whole experiment for a green diamond singleton while in the distractor condition, one of the circles was red (see Fig. 1).The key finding of the additional singleton search task is that reaction time (RT) is longer in conditions in which the additional distractor singleton is present relative to when it is absent (see Fig. 1). As is clear from the results, when the target shape and distractor color switch roles randomly throughout the experiment (the 1991 version of this task), the interference effect is much larger than when observers consistently search for the same target (e.g., a green diamond) while the same color distractor is present (a red circle) as was used in the 1992 version.

In this kind of research, RTs are typically longer when a distractor is present than when it is absent. *Attentional capture* is defined as the unintentional allocation of attention to an irrelevant distractor, despite attempts to remain focused on the task at hand. This effect is typically interpreted as evidence that attention is initially captured to the more salient color distractor, and only subsequently shifts to the target singleton, thereby delaying the response. The findings obtained with this paradigm have led to what is known as the *stimulus-driven account* (Theeuwes, 1991, 1992, 1994a, 1994b, 2010): During parallel processing across the display, the most salient item (regardless of whether it is the target or the distractor) pops out among the other display elements and captures attention. If no color distractor is present, only the target singleton pops out and captures attention and a response to the line inside the target can be given quickly (the no distractor condition). If there is a color distractor and it is more salient than the target singleton, then the distractor captures attention first. After the color distractor is rejected as not being the target, and attention is disengaged from its location by suppressing it (Theeuwes et al., 2000), attention shifts to the item that is the second most salient item in the display (which in this case is the target). Only after this additional shift of attention to the irrelevant singleton, a response can be given. The additional shift of attention to the distractor location, followed by disengagement and re-engagement with the target, increases RT relative to the no-distractor condition. This is the basic attentional capture effect. Crucially, Theeuwes (1992, Experiment 2) showed that when the color distractor is less salient than the target (e.g., an orange/greenish distractor among green nontargets), it does not capture attention. While Theeuwes (1992) showed that the less salient color singleton still popped out from the display, the pop-out from the display by the less salient distractor took longer than that of the target. Because the target and distractor were engaged in a race for attentional priority, the less salient distractor was outcompeted by the more salient target and therefore failed to capture attention. Consistent with this notion of attentional competition, Duncan and Theeuwes (2024) recently showed that when two distractors are present, attention is first captured by the most salient distractor, then shifts to the less salient distractor, and only thereafter moves to the target.

The basic findings of the additional singleton paradigms showing interference by the presence of the salient distractor have been replicated numerous times using various measures: RT (Adam et al., 2021; Bacon & Egeth, 1994; Hickey et al., 2006; Kim & Cave, 1995; Mathôt et al., 2010; Wang & Theeuwes, 2020), *d′* (Theeuwes & Chen, 2005; Theeuwes et al., 2004; van Moorselaar & Theeuwes, 2021), saccadic eye movements (Gaspelin et al., 2017; Godijn & Theeuwes, 2002; Mulckhuyse et al., 2009; Theeuwes et al., 1998, 1999), hand movements (Hunt et al., 2007), event-related potentials (Burra & Kerzel, 2013; Gaspar et al., 2016; Hickey et al., 2006; Kiss et al., 2008; Schubö, 2009), fMRI (de Fockert & Theeuwes, 2012; Richter et al., 2024), single cell recordings in monkeys (Cosman et al., 2018; Klink et al., 2023; Ogawa & Komatsu, 2004), and intracranial recordings in humans (Lin et al., 2024).

### Design features

#### The 1991 versus the 1992 version of the additional singleton task

Originally, there were two versions of the additional singleton task (see Fig. 1). In the 1991 version, observers searched for a unique shape singleton—either a diamond among circles or a circle among diamonds—while ignoring a color distractor singleton, such as a green item among red ones or vice versa. Both shape and color assignments varied randomly across trials. As is shown in Fig. 1, this version produced large interference effects of approximately 130 ms.

Later studies (Kerzel & Barras, 2016; Lamy et al., 2006; Li et al., 2023; Pinto et al., 2005) revealed that the very large interference effects were primarily due to intertrial priming. When the target and distractor switched colors between trials, interference increased significantly compared with when their colors remained the same. For instance, if the target was red on trial *n* – 1, red would be primed; if the distractor was then red on the following trial, it would capture attention much more strongly due to this priming. Importantly, further research confirmed that this priming effect occurs automatically (Hickey et al., 2010; Theeuwes et al., 2006).

In the 1992 version of the additional singleton paradigm (Theeuwes, 1992), the target and distractors remained constant throughout the entire experiment. For example, participants were asked to search for a green diamond among green circles, meaning that the target-defining feature was shape, and this never varied across trials. On distractor-present trials, one of the green circles was replaced by a red circle. Although the color singleton was completely irrelevant to the task and the target was always defined by shape, participants consistently showed attentional capture by the irrelevant red distractor.

This capture effect resulted in a modest but reliable increase in RT of about 20 to 30 ms. Importantly, this interference did not diminish even after participants completed as many as 2,000 trials. Because the target remained exactly the same on every trial, intertrial priming played no role. Participants must have formed a clear and stable search template for the green diamond, yet they were still unable to prevent their attention from being captured by the salient color distractor.

These findings are significant because they challenge purely top-down models of visual attention. Even in a task that allows for strong goal-directed control, attentional capture by a task-irrelevant but visually salient stimulus persisted. This supports Theeuwes’ stimulus-driven account of attention, which argues that certain types of salient stimuli such as color singletons automatically attract attention regardless of task goals or intentions (Theeuwes, 2010).

#### Attentional capture and disengagement

The interference effect caused by the distractor can vary from ~27 to 40 ms in typical laboratory studies (Theeuwes, 1992) to ~370 to 580 ms (Adam et al., 2024) during surprise trials when participants only receive one distractor trial during a whole experiment (see also Horstmann, 2002, 2015). The magnitude of the interference effect reflects both the extent of attentional capture by the salient singleton and the time required to disengage attention from the distractor before shifting it to the target. As claimed earlier (Theeuwes, 2010; Theeuwes et al., 2000), in some cases there will be capture but disengagement is so fast that the effect of the presence of a distractor on RT is very small or in some cases even absent. For example, Theeuwes et al. (2000) argued that if a distractor appears before the search array, as in the contingent capture paradigm of (Folk et al., 1992), the salient singleton likely captures attention; however, by the time the search array is presented, attention has already been disengaged. This may give the false impression that the distractor was ignored, when in fact it initially captured attention (see also Geng & Duarte, 2021).

An intriguing question is what determines the speed with which one can disengage attention once a salient distractor has captured it. If the salient distractor looks very different from the target, the decision to disengage attention can likely be made very quickly. However, if the distractor shares many features with the target, it may take considerably longer to determine whether it is not the target but a distractor. This is precisely what was demonstrated in a previous study by Born et al. (2011), who showed that a salient distractor dissimilar to the target produces strong capture with little or no engagement, allowing for very rapid disengagement. In contrast, a salient distractor sharing many features with the target produces both capture and strong engagement. When the distractor resembles the target, additional processing (i.e., while attention is stuck at the location of the distractor) is needed to determine whether the captured object is indeed the target or merely a distractor.

While the initial capture is entirely bottom-up and stimulus-driven, the process of determining whether the selected object is indeed the target or a distractor is very much under top-down control (Theeuwes, 2010). This implies that top-down processes determine the speed of disengagement (Theeuwes, 2010). This account can explain many previous findings. For example, in the contingent capture paradigm of Folk et al. (1992), a distractor that is a color singleton, matching the target’s defining feature, produces a reaction-time cost, whereas a distractor defined by an abrupt onset, and thus very different from the color target, produces virtually no cost. This also explains why, in Zivony and Lamy (2018), abrupt onsets that did not match the target color produced capture without engagement (see also Gaspelin et al., 2016).

#### Parallel search

The starting point of the 1991 and 1992 studies was the assumption that visual information processing involves two functionally independent stages: an early, preattentive stage that operates in parallel across the visual field without capacity limitations, and a later, capacity-limited attentive stage that can process only one or a few items at a time (e.g., Broadbent, 1958, 1982; Neisser, 1967; Treisman & Gelade, 1980). Based on this framework, the central question was whether the early parallel stage of visual processing can selectively guide the subsequent serial deployment of attention. Because this was the central question, in the original studies, display size was varied from five to nine elements and determined whether search functions were flat.

As Fig. 1 illustrates, the search functions were essentially flat, with slopes that did not differ significantly from zero. Although such patterns are typically interpreted as evidence for parallel search, it is well known that search slopes are difficult to interpret (Palmer, 1994; Townsend, 1971). What appears to be parallel processing may instead reflect a fast serial, clump-wise search strategy, in which small groups of items are searched in parallel. Notably, this clump-wise processing can also reduce the influence of a salient distractor (see also Liesefeld & Müller, 2020; Theeuwes, 2023a, b).

In a recent study, de Waard and Theeuwes (2026) employed a novel method to determine whether visual search is truly conducted in parallel or instead proceeds serially (clump-wise). They showed that attentional capture by a salient distractor occurs when search is parallel, whereas capture is absent when search is serial in clumps.

Given these findings, the conclusion from 1992 still holds: “The present study demonstrates that the parallel stage cannot selectively guide the attentive stage to just the known-to-be-relevant target feature” (Theeuwes, 1992; p. 605).

#### Distractor-present versus distractor-absent trials: Mixed versus blocked designs

In the original additional singleton experiments of 1991 and 1992, distractor-present and distractor-absent trials were presented in separate blocks. The rationale for this design was that, during a distractor-present block, participants had ample opportunity to adopt strategies to prevent capture by the distractor. Nevertheless, even under these conditions, capture still occurred. Most follow-up studies varied distractor presence–absence within blocks, usually half of the trials with a distractor while the other without a distractor. In one study, Müller et al. (2009) systematically varied distractor prevalence between 20% and 100% and found that capture was strongest when the proportion of distractor-present trials was relatively low. The most extreme case are experiments in which the distractor is presented only once (Adam et al., 2024; Horstmann, 2015; Horstmann & Ansorge, 2006). Adam et al. (2024) reported that capture by a salient singleton in such surprise trials was approximately nine to 15 times larger than what is typically observed in attentional capture studies.

These unusually large capture effects in surprise trials and the reduction of the size of capture when the distractor is less prevalent are related to the notion of selection history (Awh et al., 2012; Theeuwes, 2025). When a distractor has been encountered many times before, interference by the distractor is reduced. While one might assume that selection history prevents attentional capture by the distractor singleton altogether, it is more plausible that selection history facilitates faster disengagement of attention from the distractor. Because attention is disengaged so fast, the capture effect is relatively small. Of course, in surprise trials, following capture, participants may be so surprised by the unexpected event that disengagement is very slow.

As noted by Adam et al. (2024), a single surprise trial minimizes the influence of participants’ goals and expectations, offering a clearer and more pure measure of stimulus-driven capture. One-shot experiments are also valuable because they can offer insights into the magnitude of capture and distraction in truly unexpected everyday events—for example, when a driver suddenly encountering an unusual object on the road, like a large piece of furniture that has just fallen from a vehicle.

#### Use larger display sizes

In most early versions of the additional singleton paradigm, displays contained a relatively large number of elements, typically between five and nine (Theeuwes, 1992) and sometimes as many as 20 (Theeuwes, 2004). In more recent versions, however, displays often contain only four equally spaced elements (Adam et al., 2021; Chang & Egeth, 2019, 2021; Gaspelin et al., 2017; Gaspelin & Luck, 2018). While this design change may seem minor, it has important consequences for visual search. Previous research shows that item saliency is influenced by local feature contrast: the degree to which an item differs from its immediate neighbors (Nothdurft, 1993). When a display contains only a few items that are evenly spaced around fixation, the elements are relatively far apart. This larger spacing weakens local center–surround feature contrast: each item has fewer nearby competitors, and the nearest neighbors are more distant, so the visual system receives a less distinctive “odd-one-out” signal. As a result, neither the target singleton nor a salient distractor singleton produces a strong priority-map peak, and both appear less salient (Itti & Koch, 2001; Nothdurft, 2000). Because the target is less salient, it cannot be detected through preattentive parallel search and instead requires a more serial, clump-wise search. Likewise, the reduced salience of the distractor decreases its ability to capture attention.

The impact of reduced saliency of both target and distractor on visual search is well illustrated by two studies. In Theeuwes (2004), there was no attentional capture at a display size of five items, a small capture effect at size nine, and large capture effects at sizes 12 and 20. Similarly, Wang and Theeuwes (2020) using the additional singleton paradigm combined with a letter-probe task (Gaspelin et al., 2015) showed that for small heterogeneous displays (set size four) there was no capture, while for larger display sizes (set size ten) there was significant capture by the salient singleton.

As argued previously (Theeuwes, 2004, 2023a, 2023b, 2025) attentional capture by a salient distractor occurs when search proceeds in parallel across the display. In contrast, when the target singleton lacks sufficient salience, search must proceed in a more constrained manner, requiring the sequential discrimination of individual items or small groups of items to determine whether they match the target template. Note when the target is not salient even the most salient distractor singleton can be ignored (Stilwell et al., 2022; Stilwell & Gaspelin, 2021), simply because search for the target proceeds serially in the display.

In a recent study, de Waard and Theeuwes (2026) used a different approach to test whether visual search proceeds in parallel or serially. Rather than presenting a single target, each search display contained two targets that either required the same response (compatible) or different responses (incompatible), a method pioneered by Lee et al. (2021). The results revealed a robust two-target compatibility effect when observers performed the classic additional-singleton task, providing strong evidence for genuine parallel search. During parallel search, the salient color-singleton distractor reliably captured attention, slowing responses when present, and the strong compatibility effect indicated that multiple items were processed simultaneously. In contrast, when the display required (at least partially) serial (clump-wise) search, the color singleton produced no performance cost (and sometimes a small benefit), and compatibility effects were absent or greatly reduced, consistent with sequential item processing rather than parallel processing. It was concluded that conclude that “capture vs. no capture” is mainly determined by whether the target is salient enough to be located by parallel search. If search is conducted serially, there is no capture.

Parallel versus serial search is central to Theeuwes’s attentional window account (Belopolsky & Theeuwes, 2010; Belopolsky et al., 2007; Theeuwes, 1994a). When the target stands out from the background, the display allows observers to adopt a large attentional window that covers the entire display, enabling efficient, parallel selection but also allowing other salient singletons within the window to capture attention. When the target does not stand out, the display requires discrimination among individuated items, causing the attentional window to shrink to one or a few elements. As a result, salience computations are largely restricted to the contents of that window, reducing the influence of salient items outside it (Belopolsky & Theeuwes, 2010). Note, however, that two recent studies manipulating the size of the attentional window found no evidence that attentional capture depended on whether the salient distractor fell inside or outside that window (Ma et al., 2026; Ruthruff et al., 2026).

A recent study by Stilwell et al. (2023) used a psychophysical technique to objectively quantify salience. This approach offers a promising way forward for determining the minimum salience required for a target to be detected through parallel search and for a distractor to capture attention. There are also alternative ways to measure salience. For example, Theeuwes (1992) reported that observers took about 500 ms to find a red target among green nontargets, but about 560 ms to find a diamond target among circle nontargets. Although both targets could be detected via parallel search (search functions were flat), the shorter latencies for the color singleton indicated that it was more salient than the shape singleton.

#### Capture by the distractor singleton is purely stimulus-driven

A key design feature of the classic additional singleton task is that the distractor singleton is never the target, giving observers no incentive to attend to it. If, despite the observer’s intentions, the distractor singleton is still selected first, this provides strong evidence for stimulus-driven, bottom-up selection. As Yantis and Egeth (1999) emphasized, purely bottom-up selection can only be claimed when the capturing stimulus feature is entirely task-irrelevant, leaving no reason for the observer to attend to it deliberately. Yantis and Egeth (1999) argued: “If an object with such an attribute captures attention under these conditions, then and only then can that attribute be said to capture attention in a purely stimulus-driven fashion” (p. 663). The additional singleton task meets this criterion, establishing conditions for pure stimulus-driven capture. By contrast, in paradigms such as contingent capture, the target appears at the location of the irrelevant singleton at chance level, making the singleton partially relevant and the paradigm less suitable for isolating pure bottom-up capture. Also, although attentional capture using the classic additional singleton paradigm is typically regarded as a purely stimulus-driven effect, some have argued that capture by an irrelevant salient singleton occurs because observers adopt a top-down goal to search for any salient item (Bacon & Egeth, 1994; Leber & Egeth, 2006).

Further evidence for pure bottom-up capture comes from studies demonstrating inhibition of return (IOR) at the location of the distractor singleton. For example, using a placeholder display presented prior to the search display (similar to the contingent capture paradigm), Theeuwes and Chen (2005) included a color-distractor singleton in the placeholder display that appeared either 60 ms or 500 ms before the search display. The subsequent search display contained only a shape target singleton and no distractor singleton. Observers reported the orientation of the line segment within the shape singleton. The search display was presented briefly and then masked, and performance was assessed using sensitivity (*d′*). The results revealed a biphasic pattern at the distractor singleton’s location: Compared with the no-distractor condition, sensitivity initially increased and then decreased. This early facilitation suggests that spatial attention was involuntarily drawn to the distractor, whereas the later reduction reflects suppression at that location (Handy et al., 1999). This sequence represents a pattern associated with IOR, in which attention reflexively shifts to a location and, after disengagement, is inhibited from returning (Posner & Cohen, 1984). Using RTs, another study reported a similar pattern: Observers were slower to detect the offset of a dot when it appeared inside the color singleton than at another location (Theeuwes & Godijn, 2002). Notably, IOR does not emerge when attention is directed voluntarily in a top-down manner (Posner & Cohen, 1984).

It is interesting to notice that these earlier findings which were explained in term of the occurrence of IOR have many parallels with what in recent years has been called reactive suppression (Huang et al., 2024; Won et al., 2019). According to this notion, suppression can only occur after the object has been attended (Theeuwes, 2025). One could argue that this type of suppression is like the search-and-destroy hypothesis (Moher & Egeth, 2012), which claims that feature suppression is only possible after attending to the location of the distractor.

#### Use compound search

One key design feature of the additional singleton task is that it employs compound search (Duncan, 1985), in which observers search for a specific target (e.g., a green diamond) but respond to the orientation of a line segment inside the shape. Using a compound search allows response selection factors to be held constant across conditions, thereby isolating the effects of experimental manipulations on visual search and target identification. Consequently, any reaction-time effects associated with the presence of a color distractor can be attributed to perceptual interference rather than to response-related interference. In more classic visual search tasks (Wolfe, 1994), observers typically have to indicate whether the target is present or absent. In that case it is unclear whether RT benefits or costs are driven by differences in search efficiency or by benefits due to response selection.

To illustrate how these effects can unfold, consider a study by Theeuwes et al. (2006) in which observers performed the additional singleton task. Yet, unlike typical additional singleton tasks, observers were cued before each trial. A highly valid verbal cue would say “shape” or “color,” indicating whether the target would be a shape singleton or a color singleton. Observers responded “present” when a singleton was present or “absent” when there was no singleton. There was a clear cueing effect: Observers were faster to respond when the cue was valid than when it was invalid. A finding like this is typically interpreted as evidence that the cue allows observers to actively set themselves for the likely upcoming target. Within frameworks such as the dimensional weighting account (Müller et al., 2003) or guided search (Wolfe et al., 2003), it is assumed that observers use the advance cue to assign attentional weight to the most probable target dimension, thereby improving attentional guidance toward the target.

However, in a follow-up experiment Theeuwes et al. (2006) showed that running exactly the same procedure but replacing the target-present/target-absent response with a compound search task (responding to the line orientation inside the target) eliminated the cueing effect entirely. This finding suggests that, although cueing effects are typically interpreted as evidence that advance knowledge guides search (Found & Müller, 1996; Müller et al., 2003; Wolfe et al., 2003), they may instead reflect facilitation of responding once the target has been located. Using a compound search task makes it possible to disentangle effects of attentional guidance from those that operate at the response stage (see also Mortier et al., 2005; Starreveld et al., 2004).

While it is important to design experiments that disentangle effects on visual search from those on response processes (Prinzmetal et al., 2005), versions of the additional singleton task employing present/absent responses (van Moorselaar & Theeuwes, 2022) and *d′* measures (Theeuwes & Chen, 2005; van Moorselaar & Theeuwes, 2021) yield essentially the same pattern of results, showing capture by the salient singleton.

#### Capture of attention does not mean capture of the eyes

Although distractors can sometimes capture both attention and the eyes (Godijn & Theeuwes, 2002; Kramer et al., 1999; Theeuwes & Belopolsky, 2012; Theeuwes et al., 1998, 1999), attentional capture does not always result in an overt eye movement toward the distractor (Theeuwes et al., 2003). An eye movement to a location provides compelling evidence that attention must have been allocated there, but the absence of an eye movement provides no basis for concluding that attention was never directed there. This is especially true when capture is brief and followed by rapid disengagement, because attention can be pulled to the distractor and then released too quickly for a saccade to be programmed and executed, leaving few or no overt eye movements toward the distracting stimulus. According to the competitive integration model (Godijn & Theeuwes, 2002), a saccade will only be executed if activation in the saccade map exceeds a critical threshold; otherwise, attention may be captured without an eye movement toward the distractor.

A recent study by Klink et al. (2023) provides compelling evidence that the eyes do not always reveal where attention was captured. Using an oculomotor version of the additional singleton task in macaques, they recorded neural activity from V4 while simultaneously monitoring eye movements. By looking at the eye movements of the macaques, it appeared that the salient distractor was completely ignored: the monkeys’ eyes moved directly to the target and never fixated the distractor. Neuronal data, however, told a different story. Early in the trial, V4 responses showed enhancement at the distractor’s location, followed ~150 ms later by suppression. This demonstrates that the presence or absence of eye movements toward a distractor does not, by itself, indicate whether the distractor captured attention. In particular, the absence of eye movements does not imply the absence of attentional capture. The reverse, however, does hold: When the eyes move to a distractor’s location, attention must also have been allocated there (Deubel & Schneider, 1996; Godijn & Theeuwes, 2003).

#### Be aware of the location next to the distractor

Previous research has shown that the strongest interference, measured as the RT cost between distractor-present and distractor-absent conditions, occurs when the target singleton is positioned adjacent to the distractor singleton. For instance, Theeuwes (1992) reported that with a display size of nine, interference was about 70 ms when the target and distractor singletons were next to each other, but only about 15 ms when they were located on opposite sides of the visual field (see this analysis in Theeuwes and Godijn, 2001). Similarly, Mounts (2000) found that letter identification (*E* or *H*) was slowest when the irrelevant color singleton was positioned adjacent to the target.

There are multiple reasons why such a gradient is found. One explanation attributes it to a *suppression surround*, suggesting that the selection and subsequent suppression of the distractor singleton spreads to neighboring elements (Mounts, 2000). If the target happens to be located next to the distractor, this suppression will adversely affect its processing. This account is similar to the *biased competition view* (e.g., Desimone & Duncan, 1995), in which objects close to one another compete more strongly for neural representation than objects further apart. Similarly, inhibition is assumed to occur to prevent ambiguities in perceptual coding (Luck et al., 1997). Consistent with these viewpoints, Mathôt et al. (2010) showed that when the distance between the target and distractor singleton is kept constant, interference is greater when both are presented in the same hemifield than when they are presented in different hemifields. Finally, the target–distractor distance effects can also be explained in terms of *local feature contrast* (Nothdurft, 1993). The farther away the target is from the distractor, the more it stands out from the surrounding environment. Conversely, the closer the distractor singleton is to the target singleton, the less salient the target becomes, and the longer it takes to re-shift attention back to the target after it has been captured by the distractor. This mechanism is related to the *weight linkage* process described by Duncan and Humphreys (1989), which refers to the ease with which one is able to reject non-target elements (see also Wang & Theeuwes, 2020, for a similar explanation). Regardless of the underlying mechanism, the cleanest way to investigate attentional capture is to avoid presenting the distractor singleton directly next to the target.

#### Evidence for a shift of attention to the distractor singleton

In the additional singleton task, the presence of an irrelevant singleton reliably increases RTs compared with when it is absent (see Fig. 1). Theeuwes (2010) argued that this cost reflects attentional capture: attention is initially drawn to the distractor before shifting to the target. An alternative “filtering costs” account (Becker, 2007; Folk & Remington, 1998; Kahneman et al., 1992) proposes a different mechanism. Rather than assuming that attention is ever directed to the distractor location, it argues that attention is guided straight to the target singleton, but the presence of another singleton interferes with selection. This interference is thought to slow target processing because the visual system must engage in an effortful, time-consuming filtering operation to resolve the competition between items. From this perspective, longer RTs in the distractor condition reflect nonspatial filtering costs rather than a spatial shift of attention.

However, several lines of evidence suggest that filtering costs alone cannot explain these effects. Previous studies have employed what has been termed the *identity intrusion* technique (Schreij et al., 2008, 2010; Theeuwes, 1996; Theeuwes & Burger, 1998), in which the distractor singleton carries an identity (e.g., a letter inside the singleton) that is either congruent or incongruent with the identity placed with the target singleton. If attention is never allocated to the distractor singleton, the identity of the letter inside the distractor singleton should have no effect on performance. Yet we reported a clear congruency effect: Responses were slower when the distractor identity conflicted with the target than when it matched (Theeuwes, 1996). Such effects can only arise if attention was momentarily allocated to the distractor location, allowing its identity to be processed. These findings therefore provide strong evidence that irrelevant singletons do capture spatial attention, consistent with the stimulus-driven account and incompatible with a purely nonspatial filtering explanation (but see Folk et al., 2009, for a different interpretation).

More recent work by Gaspelin and colleagues also provides direct evidence against a filtering-cost account. In their probe-task study (Gaspelin et al., 2015, Experiment 1), probe recall at the singleton location was enhanced relative to the baseline level for nonsingleton distractors. This pattern is difficult to reconcile with a filtering-cost explanation, which would predict only a delayed decision about where to allocate attention rather than enhanced processing at the singleton location.

The earlier discussed study’s showing of IOR at the distractor location (Theeuwes & Chen, 2005) also provides evidence that the distractor captured spatial attention. IOR is widely regarded as an attentional phenomenon (Posner & Cohen, 1984), reflecting an inhibitory mechanism that prevents attention from returning to a location once it has been attended and subsequently disengaged (Klein, 2000). Thus, the very occurrence of IOR at the distractor location implies that attention was first directed there. Consistent findings have been reported in related work (Folk & Remington, 2006; Lamy & Egeth, 2003; Theeuwes & Godijn, 2002). Importantly, this evidence further rules out the filtering-cost account, since IOR can only arise when spatial attention has actually shifted to the distractor location.

#### Psychometric properties of the paradigm

In the additional singleton paradigm, attentional capture, operationalized as the RT difference between distractor-present and distractor-absent trials, shows modest reliability at the individual level. Ivanov et al. (2023) report a split-half reliability ranging from *r* = 0.51 to 0.67 and a test–retest correlation of *r* = 0.42 over a 2-month interval in an online study. Slightly higher test–retest estimates were reported in earlier laboratory studies with shorter retest intervals (e.g., Weichselbaum et al., 2018). Together, these values indicate partial temporal stability, but also substantial measurement noise (i.e., individuals who exhibit stronger attentional capture in one session tend, to a moderate extent, to do so again at retest). Notably, several studies have shown that oculomotor indices of attentional capture are more reliable than RT-based measures (Kim et al., 2024; Weichselbaum et al., 2018), often exceeding the threshold (>.70) considered acceptable for the investigation of individual differences (see Brysbaert, 2024).

The limited reliability of RT-based measures of attentional capture is compounded by inherent psychometric problems of RT difference scores more generally: Subtracting two noisy and highly correlated component measures amplifies error, removes shared variance related to general processing speed, and yields residuals with low reliability (Hedge et al., 2018). As a consequence, the use of such difference scores to index individual performance imposes a ceiling on observable correlations with other constructs and can severely compromise statistical power (see Bogaerts et al., 2025).

In summary, recent psychometric work indicates that attentional capture likely reflects a stable individual characteristic, but one that is measured with considerable error using standard RT difference-score measures. While these measures robustly characterize group-level effects, attention to task reliability and cautious interpretation is required when they are used for individual-level or predictive inferences (see also Goodhew & Edwards, 2019; Hedge et al., 2018).

## Conclusion

Below, we outline guidelines designed to maximize the utility of the paradigm and to minimize misinterpretations of attentional capture effects. These recommendations aim to ensure robust effects while helping to avoid common pitfalls. In doing so, we hope that the additional singleton task will continue to provide unique leverage in disentangling stimulus-driven selection from top-down control. It should be noted that these “do’s and don’ts” should be interpreted as my perspective on this issue, rather than a universally accepted position.

### Designing an additional singleton task: Do’s and don’ts

#### Do’s

- **Make sure the target and distractor are both salient.** Ensure that both the target and the distractor are sufficiently salient to compete. Capture occurs only when the distractor is more salient than the target (Theeuwes, 1992).
- **Use larger display sizes.** Include at least 6–10 items. Small displays (e.g., four items) reduce local feature contrast, making both targets and distractors less salient and undermining capture effects (Theeuwes, 2004; Wang & Theeuwes, 2020).
- **Employ compound search.** Require responses to a feature of the target (e.g., line orientation inside the target shape), ensuring that RT costs reflect attentional capture rather than response-selection processes (Duncan, 1985; Theeuwes et al., 2006).
- **Consider distractor prevalence.** Manipulate distractor-present versus absent trials carefully. Low prevalence or one-shot “surprise trials” maximize capture, whereas frequent distractors facilitate faster disengagement (Adam et al., 2024; Horstmann, 2015).
- **Measure capture with multiple indices.** Combine RT with neural, ERP, or eye-movement measures, as attentional capture does not always translate into overt saccades (Klink et al., 2023).
- **Acknowledgement disengagement.** Capture is stimulus-driven, but disengagement depends on top-down control and target–distractor similarity (Born et al., 2011; Theeuwes, 2010).
- **Interpret RT effects carefully.** The RT difference between distractor-present and distractor-absent trials reflects both capture and disengagement.

#### Don’ts

- **Don’t use small, sparse displays.** With too few items, search tends to be more serial, reducing distractor salience and capture effects.
- **Don’t allow the distractor to ever be the target.** If the distractor can sometimes be the target, the paradigm no longer tests pure bottom-up capture (Yantis & Egeth, 1999).
- **Don’t confuse filtering costs with capture.** Increased RTs should not automatically be attributed to filtering; converging evidence shows attention actually shifts to the distractor.
- **Don’t assume absence of eye movements means absence of capture.** Attention can be captured without an overt saccade (Godijn & Theeuwes, 2002; Klink et al., 2023).
- **Don’t use simple present/absent responses alone.** Such tasks can confound attentional guidance with response processes; compound search avoids this problem.
- **Don’t ignore selection history.** Previous target–distractor mappings strongly influence capture via intertrial priming; randomizing target/distractor assignments increases interference (Hickey et al., 2010).
- **Don’t place the distractor singleton directly next to the target.** Target–distractor adjacency produces disproportionately strong interference, complicating interpretation of capture effects (Mounts, 2000; Theeuwes, 1992).

## Data Availability

Not applicable.
